# Combining Genome-Wide Association Study and Gene-Based Haplotype Analysis to Identify Candidate Genes for Alkali Tolerance at the Germination Stage in Rice

**DOI:** 10.3389/fpls.2022.887239

**Published:** 2022-04-08

**Authors:** Song Mei, Guogen Zhang, Jing Jiang, Jingbing Lu, Fan Zhang

**Affiliations:** ^1^Institute of Crop Sciences/National Key Facility for Crop Gene Resources and Genetic Improvement, Chinese Academy of Agricultural Sciences, Beijing, China; ^2^College of Agronomy, Anhui Agricultural University, Hefei, China; ^3^National Nanfan Research Institute (Sanya), Chinese Academy of Agricultural Sciences, Sanya, China

**Keywords:** alkali tolerance, germination stage, genome-wide association study, haplotype analysis, rice

## Abstract

Salinity–alkalinity stress is one of the main abiotic factors limiting rice production worldwide. With the widespread use of rice direct seeding technology, it has become increasingly important to improve the tolerance to salinity–alkalinity of rice varieties at the germination stage. Although we have a more comprehensive understanding of salt tolerance in rice, the genetic basis of alkali tolerance in rice is still poorly understood. In this study, we measured seven germination-related traits under alkali stress and control conditions using 428 diverse rice accessions. The alkali tolerance levels of rice germplasms varied considerably during germination. *Xian*/*indica* accessions had generally higher tolerance to alkali stress than *Geng*/*japonica* accessions at the germination stage. Using genome-wide association analysis, 90 loci were identified as significantly associated with alkali tolerance. Eight genes (*LOC_Os01g12000*, *LOC_Os03g60240*, *LOC_Os03g08960*, *LOC_Os04g41410*, *LOC_Os09g25060*, *LOC_Os11g35350*, *LOC_Os12g09350*, and *LOC_Os12g13300*) were selected as important candidate genes for alkali tolerance based on the gene functional annotation and gene-CDS-haplotype analysis. According to the expression levels of *LOC_Os09g25060* (*OsWRKY76*), it is likely to play a negative regulatory role in alkali tolerance during rice germination. An effective strategy for improving rice alkali tolerance may be to pyramid alkali-tolerant haplotypes of multiple candidate genes to obtain the optimal haplotype combination. Our findings may provide valuable genetic information and expand the use of alkali tolerance germplasm resources in rice molecular breeding to improve the alkali tolerance at the germination stage.

## Introduction

Salinity–alkalinity stress is considered to be one of the most severe abiotic stresses affecting crop growth and development, resulting in crop yield reduction (Qadir et al., 2014). Rice is one of the most important food crops in the world, and nearly a third of the world’s population takes it as a staple food (Ray et al., 2013). Since the current rice yield level has reached a plateau, improving the salinity–alkalinity tolerance of rice varieties and expanding the rice planting area is an effective strategy to increase the total yield. Usually, the increase of soluble salt in the soil is referred to as “soil salinization,” and soil salinization and alkalization are always simultaneous processes (Vengosh, 2014). Recent studies have found that their effects on plants are not exactly the same, and there are differences in how plants respond to their stress (Shi and Yin, 1993). Therefore, salt stress and alkali stress are two different stresses and should be treated separately. Salt stress is mainly caused by neutral salts, including NaCl and Na_2_SO_4_, while alkali stress is primarily caused by HCO_3_^−^ and CO_3_^2−^ (Fang et al., 2021). Generally, salt stress causes ionic stress, osmotic stress, and oxidative stress to plants (Munns and Tester, 2008), while alkali stress is primarily caused by high pH (Wang et al., 2015) and may prove to be even more harmful to plants than salt stress (Yang et al., 2008). Rice is considered a salt- and alkali-sensitive crop (Cheng et al., 2007). The primary effects of saline–alkaline stress on rice are the decrease of germination rate, the inhibition or even death of seedling growth, and the decline of yield (Zhang et al., 2017). Nowadays, with rice direct seeding technology becoming more widely used, improving the salinity–alkalinity tolerance of rice varieties at the germination stage has become an important breeding target.

Salinity–alkalinity tolerance in rice is a complex trait controlled by multiple quantitative trait loci (QTLs; Liang et al., 2015). In recent years, many QTLs for salt tolerance in rice have been identified, and some salt tolerance genes, such as *SKC1* (Ren et al., 2005), *DST* (Huang et al., 2009), *OsCLC-1* (Nakamura et al., 2006), and *Saltol* (Thomson et al., 2010), have been cloned. In contrast, only a few QTLs are reported to influence alkali tolerance at the seedling stage in rice. Seven QTLs related to alkaline tolerance were detected using an F_2:3_ population derived from a cross between Caidao and WD20342 (Li et al., 2017). A rice alkali-tolerant mutant, called *alt1*, was identified and found that *alt1* negatively regulates alkali tolerance by preventing oxidative damage in rice (Guo et al., 2014). A calcium/calmodulin-dependent protein kinase *OsDMI3* can promote root elongation and improve alkaline tolerance of rice at germination stage by reducing Na^+^ and H^+^ influx in rice roots and maintaining ion balance (Ni et al., 2020).

Genome-wide association study (GWAS) has been widely used to identify associations between phenotypic traits and genotypes in many crops (Du et al., 2021; Ma et al., 2021a,b; Tekeu et al., 2021). Based on the GWAS using a core set of 208 rice germplasms, 6 and 14 SNPs associated with salt tolerance at the germination and seedling stages, and identified 22 candidate genes through haplotype analysis (Naveed et al., 2018). A major QTL (*RNC4*) related to root Na^+^/K^+^ ratio in *Geng*/*japonica* and *Xian*/*indica* accessions were identified by GWAS (Campbell et al., 2017). Yu et al. (2017) detected 25 SNPs associated with salt tolerance at the seedling stage using 295 rice accessions by GWAS. Using 295 *Geng* accessions, eight QTLs associated with alkali tolerance at the seedling stage were detected by GWAS (Li et al., 2019). Li et al. jointly conducted QTL analysis and GWAS analysis on root length at the germination stage under alkali stress using 184 RILs and 295 *Geng* accessions and identified a major QTL *qAT11* (Li et al., 2020b). However, few studies have dissected the genetic basis of the alkali tolerance between different subspecies during rice germination.

In this study, a total of 428 accessions from the 3,000 rice genomes (3 K-RG), mainly belonging to subspecies *Xian* and *Geng*, were evaluated for alkali tolerance at the germination stage (Wang et al., 2018). We used 2,949,726 SNPs filtered from the 4.8 M SNP dataset in the Rice SNP-seek Database (Alexandrov et al., 2015) to identify QTLs and candidate genes associated with alkali tolerance by GWAS. This study aimed to understand the genetic basis of alkali tolerance during germination and provide favorable resources of genes and germplasms for molecular breeding for alkali tolerance improvement of rice.

## Materials and Methods

### Plant Materials and Alkali Tolerance Evaluation

We used 428 accessions from the 3 K-RG to evaluate the alkali tolerance of rice at the germination stage. The accessions contained 125 *Geng* accessions, 278 *Xian* accessions, 13 *Aus*, 6 *Basmati* and 6 *admix* accessions (Supplementary Table 1). A total of 120 seeds of each accession were dried at 50°C for 3 days to make them dry completely and break their dormancy. To disinfect the seeds, they were soaked in 3% NaClO solution for 30 min, and then the seeds were washed three times with sterile distilled water before testing. For each rice accession, 20 seeds were placed into a 90-mm-diameter Petri dish with filter paper. Three replicates were conducted under control (distilled water) and alkali stress conditions (0.15% Na_2_CO_3_ solution), respectively. As a control, 10 ml of distilled water was added to each Petri dish, and 10 ml of 0.15% Na_2_CO_3_ solution was added to each Petri dish as alkali stress. After that, the seeds were grown in a growth chamber under a 14-h light/10-h dark photoperiod (28°C/26°C) with 70% relative humidity for 7 days. Throughout the test, the solution was changed regularly every day.

Seeds were considered germinated when the root length was equal to the seed length and the shoot length was equal to half of the seed length. The germinated seeds were counted each day to calculate the germination rate (GR), germination energy (GE), and germination index (GI). We used the seed germination rates on day 3 and day 7 as GE and GR (GE or GR = N_t_/N_0_, where N_t_ represents the number of germinated seeds at day *t* and *N*_0_ represents the total number of experimental seeds), respectively. The GI was calculated as follows: GI = ∑(G_t_/T_t_), where G_t_ is the accumulated number of the germinated seeds on day *t* and *T_t_* is the time corresponding to G_t_ in days (Wang et al., 2010). The mean germination time (MGT) was calculated by using the formula: MGT = ∑T_t_N_t_/∑N_t_, where N_t_ is the number of newly germinated seeds on day *t* (Alvarado et al., 1987). On day 7, the root length (RL) and shoot length (SL) of eight seeds randomly selected from each replicate were measured in both alkali stress and control conditions. Vigor index (VI) = (mean SL) × GI. We also used the ratios of the seven germination-related traits under alkali stress to the control for evaluating the response of rice accessions to alkali stress. The average of three replicates for each trait was used for data analyses.

### Genome-Wide Association Mapping

The 3K-RG 4.8 M SNP dataset was downloaded from the Rice SNP-Seek Database (Alexandrov et al., 2015).^1^ SNPs with the missing rate < 20% and minor allele frequencies >5% were filtered with PLINK (Purcell et al., 2007). Finally, a total of 2,942,166, 2,091,233, and 1,218,609 SNPs were used for GWAS in the whole, *Xian* and *Geng* panel populations, respectively. The GWAS was performed with EMMAX (Kang et al., 2010) based on a mixed linear model (MLM) to detect the associations between SNP and the alkali tolerance-related traits. The kinship matrix was generated using an identical-by-state matrix (with the “emmax-kin -v -h -d 10” parameter in EMMAX) based on the pruned subset of genome-wide SNP data (with the “indep-pairwise 50 10 0.1” parameter in PLINK) to account for the relatedness among accessions. For controlling population structure, the eigenvectors of the kinship matrix were calculated using GCTA (with the “-make-grm” parameter; Yang et al., 2011) and the first three principal components were used as covariates. The effective number of independent markers (N) was calculated with the GEC software (Li et al., 2012) and suggestive significance thresholds of association by the Bonferroni correction method (1/N) were calculated for claiming significant SNPs for the whole population (*p* = 2.15E-06), *Xian* (*p* = 2.76E-06) and *Geng* (*p* = 8.31E-06) subpopulations, respectively. Manhattan and quantile–quantile (Q–Q) plots of GWAS were created by the R package “qqman” (Turner, 2014). Based on the previously reported linkage disequilibrium (LD) decay in the 3 K-RG (Wang et al., 2018), the significant SNPs within a 300-kb region were considered a locus. A lead SNP in a locus was defined as the SNP with the lowest value of *p*, and the other significant SNPs within 150 kb on either side of the lead SNP were merged.

### Analysis of Candidate Genes

We shortlisted potential candidate genes for alkali tolerance when they met at least one of the following criteria: (1) genes with significant SNPs and functional annotation related to abiotic stress based on the Nipponbare reference genome IRGSP 1.0 (Kawahara et al., 2013) and the funRiceGenes database (Yao et al., 2018); (2) genes harboring SNPs significantly associated with more than three traits; (3) genes containing SNPs of the most significant association (*p* < 0.05/effective SNP number) with each trait. In the case of an SNP located in the intergenic region, the downstream gene was chosen. LDBlockshow (Dong et al., 2021) was used to estimate the local LD block region containing each candidate gene. The haplotype analysis was performed on all candidate genes using all SNPs within the gene coding sequence region, in which synonymous SNPs were ignored (merged in one haplotype; Zhang et al., 2021). There were at least 10 rice accessions in each haplotype. Duncan’s multiple range post-hoc tests were completed with the “agricolae” package (de Mendiburu, 2021) in R to compare phenotypic differences among the haplotypes (*n* ≥ 10 rice accessions). The expression profiles of candidate genes in Nipponbare were obtained from the RiceXPro database (Yutaka et al., 2013).

### RNA Extraction and qRT-PCR Analysis

For detecting expression levels of candidate genes, we screened two representative accessions (one alkali-tolerant and one alkali-sensitive) from each subspecies. After 24 h of alkali stress with 0.15% Na_2_CO_3_, 100 seed embryos were sampled under alkali stress and control conditions, respectively. Total RNA was extracted from germinating seeds using plant RNA extraction kit (Tiangen Biotechnology) and reverse transcribed using reverse transcription kit (Invitrogen). Real-time qRT-PCR analyses were conducted with Taq Pro Universal SYBR qPCR Master Mix (Vazyme, Q712-02). All primers used for qRT-PCR are listed in Supplementary Table 2. The relative expression levels were calculated using the 2^-∆∆CT^ method (Livak and Schmittgen, 2002).

### Statistical Analysis

We used one-way ANOVA to test the differences between subpopulations in the traits related to alkali tolerance by the “agricolae” (de Mendiburu, 2021) package in R. Spearman’s correlation coefficients between traits were calculated using the “corrplot” package in R. Box plots for displaying the trait distribution and bar charts for illustrating haplotype frequency were generated using the “ggplot2” (Ginestet, 2011) package in R.

## Results

### Phenotypic Variations in the Alkali Tolerance of Rice at the Germination Stage

Seven traits including GR, GI, MGT, VI, RL, SL, and GE were measured for 428 rice accessions under alkali stress with 0.15% Na_2_CO_3_ (named “TraitName+S”) and control condition with distilled water (named “TraitName+C”) at the germination stage (Table 1). Furthermore, we calculated the ratios of these traits under alkali stress and control conditions (named “R + TraitName”) in order to determine the degree of alkali damage (Supplementary Table 1). Under alkali stress and control conditions, the germination-related traits were distributed continuously, suggesting that multiple loci may be involved in controlling these traits (Supplementary Figure 1). We observed 12 accessions with GRS of 1.00, including eight *Xian* accessions, three *Geng* accessions, and an *Aus* accession (Supplementary Table 1). Among the four accessions with the shortest MGTS, all were *Xian* accessions. In addition, we found some accessions exhibited extreme phenotypes in other germination-related traits, such as “Lumaozhan” with the highest GES (0.90) and GIS (1.04), “Heimangdao” with the longest SLS (6.03 cm), “ARC 15091” with the longest RLS (4.53 cm), and “NCS 766” with the highest VIS (6.17).

**Table 1:** Summary of the 21 traits measured in this study.

Trait	Full names of traits	Range	Mean	SD	CV
MGTS	Mean germination time under alkali stress (d)	3.000–7.000	4.337	0.698	0.161
MGTC	Mean germination time under control condition (d)	3.000–5.556	3.785	0.434	0.115
GRS	Germination rate under alkali stress	0.017–1.000	0.647	0.266	0.411
GRC	Germination rate under control condition	0.800–1.000	0.955	0.048	0.050
GIS	Germination index under alkali stress	0.007–1.040	0.471	0.244	0.518
GIC	Germination index under control condition	0.343–1.093	0.809	0.136	0.168
VIS	Vigor index under alkali stress	0.013–6.173	1.966	1.230	0.625
VIC	Vigor index under control condition	1.235–8.016	4.323	1.160	0.268
RLS	Root length under alkali stress (cm)	0.163–4.525	1.582	0.679	0.429
RLC	Root length under control condition (cm)	1.600–12.821	6.858	2.215	0.323
SLS	Shoot length under alkali stress (cm)	1.363–6.025	3.887	0.802	0.206
SLC	Shoot length under control condition (cm)	3.018–8.383	5.293	0.791	0.150
GES	Germination energy under alkali stress	0.000–0.900	0.128	0.207	1.619
GEC	Germination energy under control condition	0.000–1.000	0.329	0.316	0.963
RMGT	Ratio of mean germination time under alkali stress to normal condition	0.735–2.004	1.149	0.158	0.138
RGR	Ratio of germination rate under alkali stress to normal condition	0.017–1.118	0.678	0.277	0.409
RGI	Ratio of germination index under alkali stress to normal condition	0.008–1.127	0.574	0.266	0.463
RVI	Ratio of vigor index under alkali stress to normal condition	0.004–1.232	0.441	0.227	0.514
RRL	Ratio of root length under alkali stress to normal condition	0.013–1.406	0.264	0.170	0.645
RSL	Ratio of shoot length under alkali stress to normal condition	0.371–1.212	0.733	0.100	0.136
RGE	Ratio of germination energy under alkali stress to normal condition	0.000–1.500	0.211	0.283	1.338

Range, Mean, SD, and CV indicate the range from minimum value to maximum value, mean value, standard deviation, and coefficient of variation of a trait, respectively.

**Figure 1:**
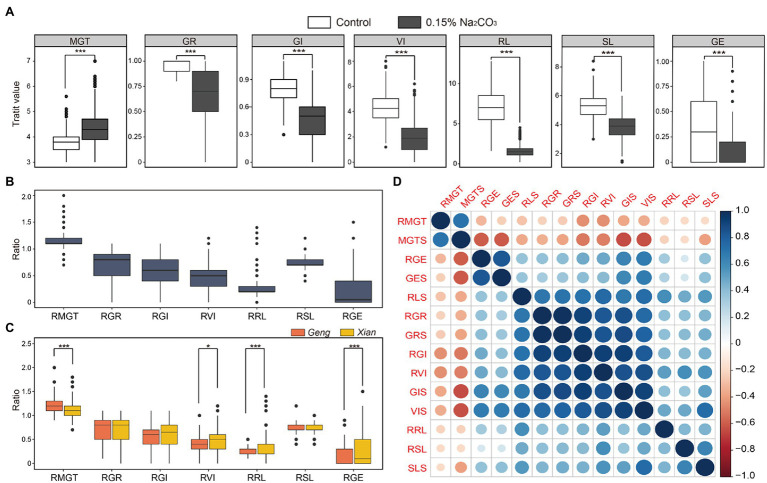
Phenotypic variations of germination-related traits under alkali stress and control conditions. **(A)** Distribution of mean germination time (MGT), germination rate (GR), germination index (GI), vigor index (VI), root length (RL), shoot length (SL), and germination energy (GE) under control and alkali stress (0.15% Na_2_CO_3_). Distribution of relative alkali damage of measured traits in the whole population **(B)**, *Xian* and *Geng* subpopulations **(C)**. **(D)** Correlation coefficient of alkali tolerance-related traits in the whole population. * and *** indicate significant difference at *p* < 0.05 and *p* < 0.001 (two-tailed Student’s *t*-test) in **(A,C)**, respectively.

In comparing the germination-related traits under alkali stress and control conditions, we found that alkali stress significantly impaired the seed germination and growth, extending germination time and reducing GE, GR, GI, VI, RL, and SL (Figure 1A). GE was the most strongly affected by alkali stress among the seven traits. On average, MGT was delayed by 14.9% under alkali stress, and GR, GI, VI, RL, SL, and GE decreased by 32.2%, 42.6%, 55.9%, 73.6%, 26.7%, and 78.9%, respectively. We used the ratio of the trait value under alkali stress to control to determine the extent of damage caused by alkali. Accordingly, alkali damage on these germination-related traits followed the following order: GE > RL > VI > GI > GR > SL > MGT (Figure 1B). Furthermore, the *Xian* subpopulation (*n* = 278) exhibited significantly higher RVI, RRL, RGE, but significantly shorter RMGT than the *Geng* subpopulation (*n* = 125; Figure 1C), suggesting that *Xian* accessions are generally more alkaline tolerant than *Geng* accessions.

According to the correlation analysis among the germination-related traits in the whole population, MGTS and RMGT were negatively correlated with the other traits, whereas GRS, RGR, GIS, RGI, VIS, and RVI demonstrated a strong positive correlation with one another (Figure 1D). In addition, correlation analysis results in the subpopulations *Xian* and *Geng* were similar to the results in the whole population (Supplementary Figure 2). These findings indicate that it is valuable to identify the loci associated with these germination traits and corresponding favorable alleles in molecular breeding for rice alkali tolerance.

### Identification of Loci Associated With Alkali Tolerance

We conducted a GWAS based on the mixed linear model for the traits related to alkali tolerance (Supplementary Figures 3–8). Using a Bonferroni correction based on the effective numbers, the genome-wide significant value of *p* thresholds were set at 2.15E-06, 2.76E-06, and 8.31E-06 for the whole population, *Xian* subpopulation, and *Geng* subpopulation, respectively. Consequently, 271, 51, and 503 SNPs significantly associated with alkali tolerance at the germination stage were identified in the whole population, *Xian* subpopulation and *Geng* subpopulation, respectively. A total of 18 significantly associated SNPs were detected both in the whole population and *Xian* subpopulation (Supplementary Figure 9A). However, there were no overlaps between the identified SNPs in *Xian* subpopulation and those in *Geng* subpopulation. By analyzing the genes in which the significantly associated SNPs were detected, it was found that there was also no overlap between the genes identified in *Xian* subpopulation and those in *Geng* subpopulation (Supplementary Figure 9B). The results suggested that the genetic mechanism of alkali tolerance in subpopulations *Xian* and *Geng* may be different. We merged the adjacent SNPs around 300 kb as a locus to reduce the redundancy in association signals of different traits. Consequently, a total of 90 loci involved in 124 associations between 106 lead SNPs and 14 traits were identified in the whole population or at least one of the two subpopulations (Supplementary Table 3).

**Figure 2:**
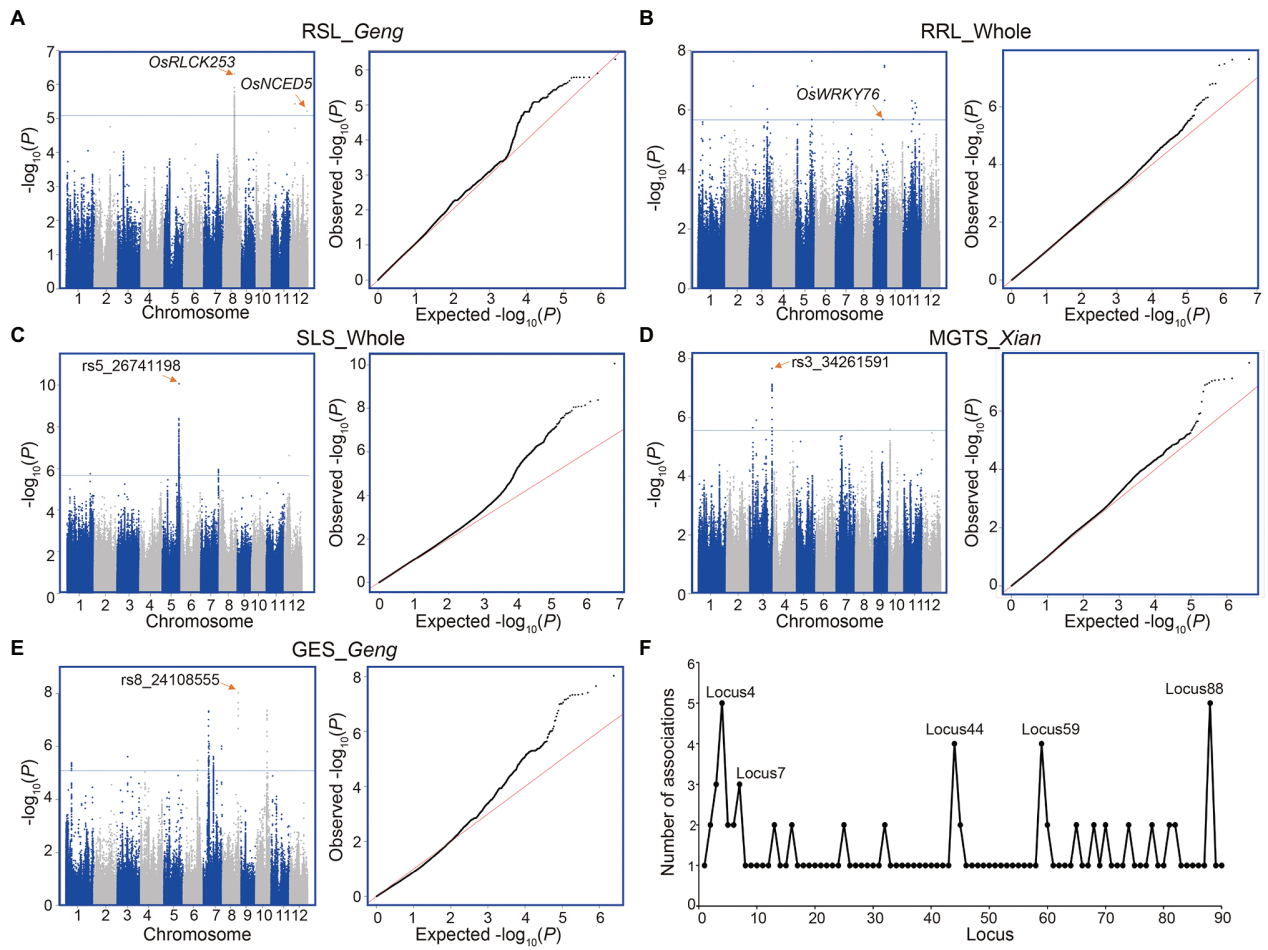
Genome-wide association study of five alkali tolerance-related traits. **(A)** RSL in the *Geng* subpopulation. **(B)** RRL in the whole population. **(C)** SLS in the whole population. **(D)** MGTS in the *Xian* subpopulation. **(E)** GES in the *Geng* subpopulation. **(F)** Number of associated traits for each of all detected loci (Supplementary Table 3). Manhattan plot (left) and Q–Q plot (right) for each panel in **(A–E)**. Horizontal lines indicate in the Manhattan plots indicate the genome-wide suggestive thresholds. The names of candidate genes or lead SNPs are shown above the corresponding association signals.

According to the annotations of the genes with identified SNPs, no reported genes for alkaline tolerance were identified. However, we found two known salt tolerance genes (*OsRLCK253* and *OsNCED5*) harboring lead SNPs (rs8_17550068 with *p* = 4.92E-07 and rs12_26284167 with *p* = 6.1E-06) associated with RSL in *Geng* subpopulation (Figure 2A). Moreover, a WRKY transcription factor gene *OsWRKY76* was significantly associated with RRL in the whole population (Figure 2B). In addition, we identified several new loci with strong associations with alkali tolerance (Figures 2C–E; Supplementary Table 3). For example, a lead SNP rs5_26741198 was significantly associated with SLS (*p* = 8.68E-11) in the whole population. The lead SNP of rs3_34261591 showed the strongest association signal (*p* = 2.15E-8) with MGTS in *Xian* subpopulation. The lead SNP rs8_24108555 with the most significant association (*p* = 9.42E-9) for GES was identified in *Geng* subpopulation (Figures 2C–E).

A comprehensive analysis was carried out concerning the 90 alkali tolerance loci, each of which was associated with one to five traits (Figure 2F; Supplementary Table 3). For example, locus 4 was associated with GRS, RLS, RGR, and RGI in the whole population and with RLS in *Geng* subpopulation. Locus 7 was associated with GRS, RGR, and RVI in the whole population. Locus 44 was associated with GES, RGE and RRL in the whole population and with RRL in *Xian* subpopulation. Locus 59 was associated with GRS and RGR both in the whole population and *Xian* subpopulation. Locus 88 was associated with GIS, VIS, SLS, and RVI in the whole population and with RSL in *Geng* subpopulation.

### Haplotype Analyses of the Candidate Genes

**Figure 3:**
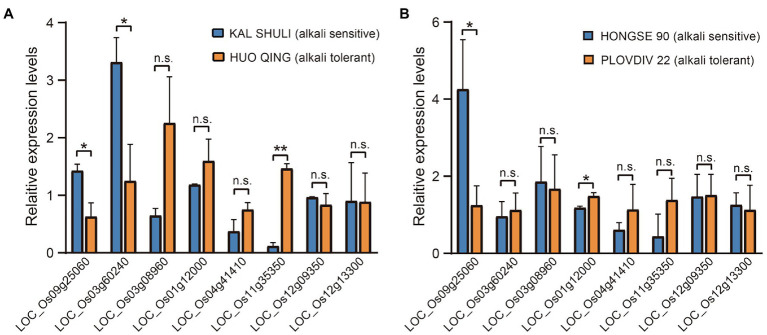
Relative expression levels of candidate genes under alkali stress for 24 h. **(A)** In two representative *Xian* germplasms. **(B)** In two representative *Geng* accessions. Gene expression was normalized to that of the *OsActin* gene control. The relative expression levels were represented by fold change relative to the expression levels of the candidate genes. ^*^p < 0.05 and ^**^p < 0.01 (two-tailed Student’ *t*-test). Data represent means ± SD (*n* = 3).

We selected 17 genes as candidate genes at 15 loci (Supplementary Table 4; see Methods for detail), and conducted a gene-CDS-haplotype analysis to determine whether there were significant differences in alkali tolerance between different haplotypes of each gene. Finally, eight genes with significant phenotypic differences between haplotypes were identified as important candidate genes for alkali tolerance, including *LOC_Os09g25060*, *LOC_Os03g60240*, *LOC_Os03g08960*, *LOC_Os01g12000*, *LOC_Os04g41410*, *LOC_Os11g35350*, *LOC_Os12g09350*, and *LOC_Os12g13300*. Based on qRT-PCR analysis, the expression levels of *LOC_Os09g25060* and *LOC_Os03g60240* were both higher in the alkali-sensitive *Xian* accession than in the alkali-tolerant *Xian* accession. On the contrary, *LOC_Os11g35350* was expressed more strongly in the alkali-tolerant *Xian* accession compared to the alkali-sensitive *Xian* accession. There were no significant differences in the expression levels of the other five genes between the two *Xian* accessions (Figure 3A). For the two *Geng* accessions, the expression level of *LOC_Os09g25060* in alkali-sensitive accession was also significantly higher than that in alkali-tolerant accession, while *LOC_Os01g12000* was expressed more strongly in the alkali-tolerant accession than in alkali-sensitive accession (Figure 3B).

**Figure 4:**
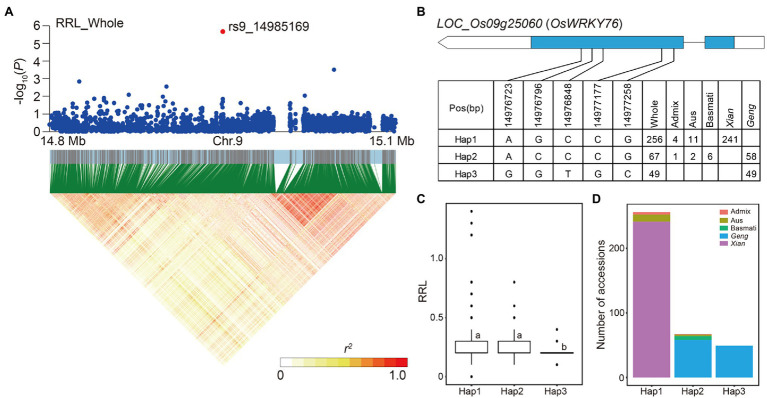
Candidate gene analysis of locus 71 on chromosome 9. **(A)** Local Manhattan plot (top) and LD heat map (bottom) of locus 71 for RRL in the whole population. The red dot indicates the lead SNP rs9_14985169 and the position of candidate gene *LOC_Os09g25060*. **(B)** CDS-haplotypes of *LOC_Os09g25060*. **(C)** The distribution of RRL in the whole population for the three haplotypes of *LOC_Os09g25060*. Different letters above each boxplot indicate significant differences among haplotypes according to Duncan’s multiple range *post-hoc* test ( *p* < 0.05). **(D)** Frequency of three haplotypes of *LOC_Os09g25060* in subpopulations.

For locus 71 on chromosome 9, the LD block region was predicted from 14.94 to 14.99 Mb (48.93 kb) and included 491 SNPs (Figure 4A). *LOC_Os09g25060* (*OsWRKY76*) had a lead SNP rs9_14985169 (*p* = 2.09E-06) for RRL in the whole subpopulation, which is a member of the rice WRKY transcription factor gene family (Ross et al., 2007). Three major haplotypes of *LOC_Os09g25060* were detected based on five SNPs in the coding region shared by at least 10 accessions (Figure 4B). Hap1 is predominant in *Xian* subpopulation and Hap2 is prevalent in *Geng* subpopulation (Figure 4D). In comparison of the RRL across the three haplotypes, Hap1 and Hap2 had a significantly higher RRLs of 0.28 and 0.26 than Hap3, which had a mean RRL of 0.19 in the whole population (Figure 4C).

**Figure 5:**
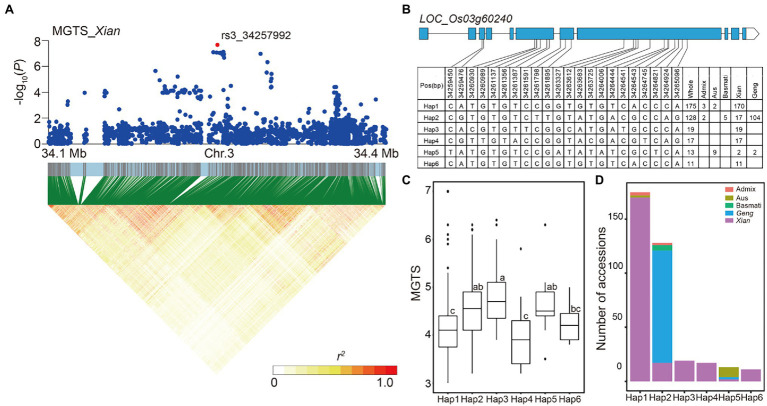
Candidate gene analysis of locus 32 on chromosome 3. **(A)** Local Manhattan plot (top) and LD heat map (bottom) of locus 32 for MGTS in the *Xian* subpopulation. The red dot indicates the lead SNP rs3_34257992 and the position of candidate gene *LOC_Os03g60240*. **(B)** CDS-haplotypes of *LOC_Os03g60240*. **(C)** The distribution of MGTS in the whole population for the six haplotypes of *LOC_Os03g60240*. Different letters above each boxplot indicate significant differences among haplotypes according to Duncan’s multiple range *post-hoc* test ( *p* < 0.05). **(D)** Frequency of six haplotypes of *LOC_Os03g60240* in subpopulations.

The LD block region for locus 32 on chromosome 3 was predicted from 34.256 to 34.267 Mb (11.16 kb) and contained 37 SNPs (Figure 5A), including the alkali tolerance candidate gene *LOC_Os03g60240* that had the most significant SNP rs3_34257992 (*p* = 7.96E-08) for MGTS in *Xian* subpopulation. Using 22 SNPs (including five non-synonymous SNPs) in the coding region of *LOC_Os03g60240*, six haplotypes were detected, each of which was shared by at least 10 accessions (Figure 5B). In the whole population, Hap4 had the lowest mean MGTS (3.98). Thus, we defined Hap4 as the favorable haplotypes of alkali tolerance, which were carried only by *Xian* accessions (Figures 5C,D).

**Figure 6:**
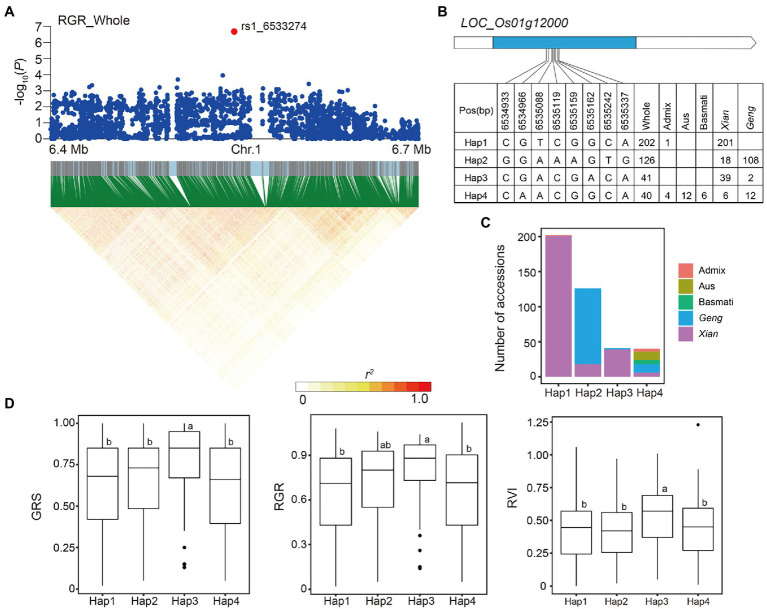
Candidate gene analysis of locus 7 on chromosome 1. **(A)** Local Manhattan plot (top) and LD heat map (bottom) of locus 7 for RGR in the whole population. The red dot indicates the lead SNP rs1_6533274 and the position of candidate gene *LOC_Os01g12000*. **(B)** CDS-haplotypes of *LOC_Os01g12000*. **(C)** Frequency of four haplotypes of *LOC_Os01g12000* in subpopulations. **(D)** The distribution of GRS, RGR, and RVI in the whole population for the four haplotypes of *LOC_Os01g12000*. Different letters above each boxplot indicate significant differences among haplotypes according to Duncan’s multiple range *post-hoc* test ( *p* < 0.05).

For locus 7 on chromosome 1, the most significant association peak for RGR, GRS, and RVI in the whole population, was predicted from 6.47 to 6.56 Mb (85.5 kb) and included 730 SNPs based on local LD analysis (Figure 6A). One of these SNPs, rs1_6533274, located in the intergenic region of two genes (*LOC_Os01g11990* and *LOC_Os01g12000*), was significantly associated with RGR, GRS, and RVI. We identified four haplotypes (*n* ≥ 10 accessions) based on eight SNPs in the coding region of *LOC_Os01g12000*, the downstream gene of rs1_6533274 (Figure 6B). Among the haplotypes, Hap1 and Hap3 were both enriched in *Xian* subpopulation, while Hap3 had significantly higher GRS, RGR, and RVI than the other haplotypes (Figures 6C,D).

**Figure 7:**
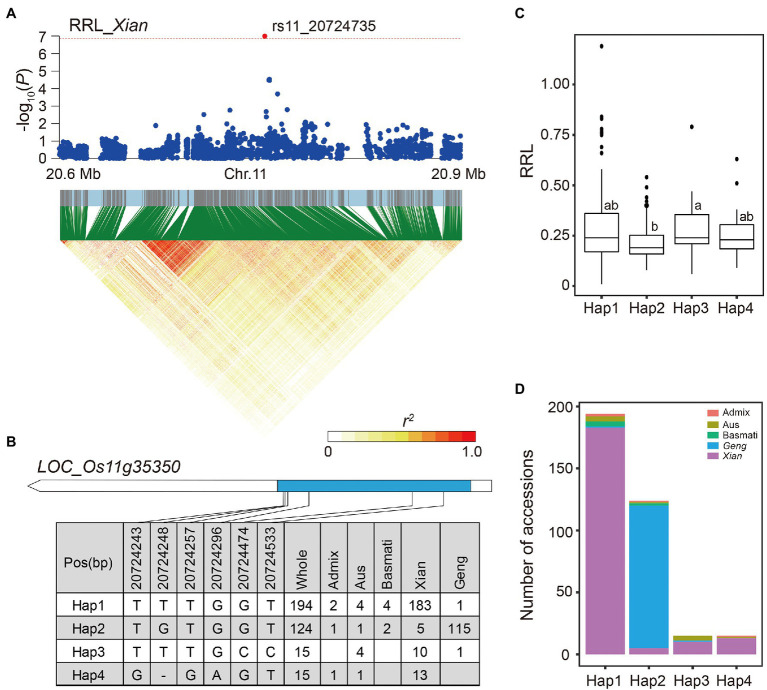
Candidate gene analysis of locus 82 on chromosome 11. **(A)** Local Manhattan plot (top) and LD heat map (bottom) of locus 82 for RRL in the *Xian* subpopulation. The red dot indicates the lead SNP rs11_20724735 and the position of candidate gene *LOC_Os11g35350*. **(B)** CDS-haplotypes of *LOC_Os11g35350*. **(C)** The distribution of RRL in the whole population for the four haplotypes of *LOC_Os11g35350*. Different letters above each boxplot indicate significant differences among haplotypes according to Duncan’s multiple range *post-hoc* test ( *p* < 0.05). **(D)** Frequency of four haplotypes of *LOC_Os11g35350* in subpopulations.

The LD block region of locus 82 was estimated to be from 20.71 to 20.75 (38.1 kb) on chromosome 11 including 438 SNPs. The SNP rs11_20724735, located in the candidate gene *LOC_Os11g35350*, was significantly associated with RRL in the *Xian* subpopulation (Figure 7A). Using six SNPs within the coding region of *LOC_Os11g35350*, four major haplotypes (*n* ≥ 10 accessions) were identified (Figure 7B). Hap3 had significantly higher RRL, which were mainly present in the *Xian* accessions compared to the other haplotypes (Figures 7C,D).

### Optimal Alkali-Tolerant Haplotype Combination

**Figure 8:**
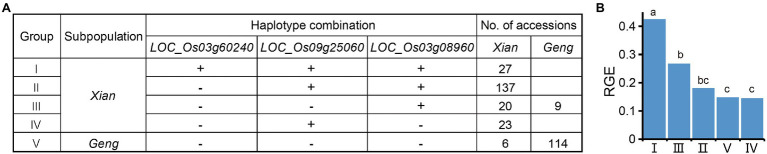
Optimal alkali-tolerant haplotype combination of candidate genes. **(A)** Combined haplotypes of *LOC_Os03g60240*, *LOC_Os09g25060*, and *LOC_Os03g08960*. “+” and “−” represent favorable and inferior haplotypes, respectively. **(B)** Comparison of the RGE among different haplotype combinations. Different letters above each histogram indicate significant differences at *p* < 0.05 (Least Significant Difference test).

Since RGE was most sensitive to alkali stress, it was used to identify the favorable haplotype of each candidate gene. As a result, Hap1 of *LOC_Os09g25060*, Hap3 of *LOC_Os01g12000*, Hap5 of *LOC_Os12g13300*, Hap4 of *LOC_Os03g60240*, Hap3 of *LOC_Os04g41410*, Hap6 of *LOC_Os12g09350*, Hap4 of *LOC_Os11g35350*, and Hap1 of *LOC_Os03g08960* were detected as alkali-tolerant haplotypes. We used a combined haplotype analysis in order to determine the breeding potential of candidate genes for alkali tolerance. There remained five groups comprising three candidate genes after the removal of rare haplotype combinations (*n* < 15 accessions). The Groups I, II, III, and IV were enriched in the *Xian* subpopulation, whereas Group V was mainly found in the *Geng* subpopulation, which showed clear *Xian*-*Geng* differentiation (Figure 8A). Compared to the other four groups, Group I containing the alkali-tolerant haplotypes at *LOC_Os03g60240*, *LOC_Os09g25060*, and *LOC_Os03g08960* had a significantly higher RGE (Figure 8B). Furthermore, the more favorable haplotypes of these candidate genes were pyramided in rice, the stronger the alkali tolerance. The results suggest that developing a pyramid of multiple favorable haplotypes/alleles for alkali tolerance may be an effective strategy for improving rice alkali tolerance at the germination stage.

## Discussion

### *Xian* Is More Alkali-Tolerant Than *Geng* During Germination

The alkali stress of 0.15% Na_2_CO_3_ can significantly inhibit rice germination (Figure 1A). Qi et al. (Qi et al., 2005, 2006) proposed the primary method to evaluate the alkali tolerance during rice germination is to measure GR, GI, and relative alkali damage rate, and they reported a wide variation range and reasonable distribution of GR, GE and their relative alkali damage rate under 0.10% and 0.20% Na_2_CO_3_ condition. In our study, GE was the most sensitive trait of the seven germination-related traits measured under alkali stress (Figure 1B), which is consistent with previous studies (Lv et al., 2013). Furthermore, the alkali tolerance levels of rice germplasms also varied considerably during germination in this study (Supplementary Figure 1). *Xian* accessions had shorter RMGT and higher RVI, RRL, and RGE than *Geng* accessions, indicating that *Xian* accessions had generally higher tolerance to alkali stress than *Geng* accessions at the germination stage (Figure 1C). This is contrary to the previous findings by Shi et al. regarding salt tolerance in rice during germination (Shi et al., 2017), inferring different mechanisms for salt and alkali tolerance in rice.

### Genetic Mechanism Underlying Rice Alkali Tolerance Differs From Salt Tolerance During Germination

Two salt tolerance-related genes *OsRLCK253* and *OsNCED5* were associated with alkali tolerance at the germination stage in this study. *OsRLCK253*, which encodes a receptor-like cytoplasmic kinase, can improve the water-deficit and salt tolerance in transgenic *Arabidopsis* plants by affecting several common endogenous gene expressions (Giri et al., 2011). *OsNCED5*, encoding a 9-cis-epoxycarotenoid dioxygenase, positively regulates ABA level, enhances salt tolerance, and accelerates leaf senescence in rice (Xiong et al., 2014). In both *OsRLCK253* and *OsNCED5*, however, there were no significant differences in alkali tolerance between different haplotypes. In addition, no cloned genes with large effects on salt tolerance were detected in this study, further supporting the perspective that different genetic mechanisms may regulate alkali tolerance and salt tolerance in rice (Sun et al., 2021).

The expression levels of candidate gene *LOC_Os09g25060* were significantly higher in alkali-sensitive accessions compared with alkali-tolerant accessions in both *Xian* and *Geng* subpopulations, suggesting its negative regulatory role in alkali tolerance during rice germination. *LOC_Os09g25060*, encoding a WRKY transcription factor *OsWRKY76*, is downregulated in the roots of Dongdao-4 in response to saline–alkaline stress (Li et al., 2020a). Previous studies have shown that the genes associated with jasmonic acid (JA) biosynthesis and JA signaling are enhanced in the knockout plants of both *OsWRKY62* and *OsWRKY76* (Liang et al., 2017). Moreover, salt stress can trigger the activation of the JA signaling pathway and inhibit the cell elongation in the elongation zone of root (Valenzuela et al., 2016). Based on haplotype analysis of *OsWRKY76*, Hap1 and Hap2 had higher RRL than Hap3 (Figure 4C), suggesting that *OsWRKY76* may affect root development by activating the JA signaling pathway, which ultimately affects rice alkali tolerance at the germination stage.

### Breeding Application of the Favorable Haplotypes

Rice can alleviate the damage of alkali stress through osmotic regulation, ion balance, antioxidant protection, and hormone regulation (Fang et al., 2021). The alkali tolerance of rice at the germination stage is a complex trait, which is controlled by multiple genes.

Among 90 loci for alkali tolerance in this study, only five loci (5.6%) were also detected in previous QTL mapping studies (Supplementary Table 3), primarily because most reported QTLs for alkali tolerance were identified at the seedling stage. In other words, the genetic mechanisms of alkali tolerance in rice may partially differ between the seedling and germination stages. Our results indicated that rice germplasms with more favorable haplotypes on candidate genes had better alkali tolerance (Figure 8), which suggests that developing an optimal haplotype combination through pyramiding multiple favorable haplotypes should be an effective strategy for improving alkali tolerance during rice germination. In addition, it is possible, through inter-subspecific crosses in rice breeding programs, to make introgression of favorable haplotypes specific in the *Xian* subpopulation into the *Geng* subpopulation to provide important donor resources for improving the alkali tolerance of the *Geng* subpopulation. For example, rice breeders may use marker-assisted selection for Hap4 of *LOC_Os03g60240* in the progeny of inter-subspecific crosses to shorten germination time under alkali stress. Thus, the candidate genes associated with alkali tolerance and their favorable haplotypes will be useful to rice alkali-tolerant breeding.

## Data Availability Statement

The original contributions presented in the study are included in the article/Supplementary Material, and further inquiries can be directed to the corresponding author.

## Author Contributions

FZ conceived and designed the experiments. SM analyzed the data and wrote the manuscript. SM, GZ, JJ, and JL performed the experiments. All authors contributed to the article and approved the submitted version.

## Funding

This work was supported by the Key Research and Development Project of Hainan Province (ZDYF2021XDNY171), and the Agricultural Science and Technology Innovation Program and the Cooperation and Innovation Mission (CAAS-ZDXT202001).

## Conflict of Interest

The authors declare that the research was conducted in the absence of any commercial or financial relationships that could be construed as a potential conflict of interest.

## Publisher’s Note

All claims expressed in this article are solely those of the authors and do not necessarily represent those of their affiliated organizations, or those of the publisher, the editors and the reviewers. Any product that may be evaluated in this article, or claim that may be made by its manufacturer, is not guaranteed or endorsed by the publisher.

## Supplementary Material

The Supplementary Material for this article can be found online at: https://www.frontiersin.org/articles/10.3389/fpls.2022.887239/full#supplementary-material

## Footnotes

^1^ http://snp-seek.irri.org/

## Abbreviations

3K-RG: 3,000 Rice genomes
MGT: Mean germination time
GR: Germination rate
GI: Germination index
VI: Vigor index
RL: Root length
SL: Shoot length
GE: Germination energy
ANOVA: Analysis of variance
GWAS: Genome-wide association study
MAF: Minor allele frequency
LD: Linkage disequilibrium
Chr: Chromosome
Kb: Kilobyte
Mb: Megabyte
SNP: Single nucleotide polymorphism
QTL: Quantitative trait locus/loci
Hap: Haplotype
