# Supplementary material for: Combining Genome-Wide Association Study and Gene-Based Haplotype Analysis to Identify Candidate Genes for Alkali Tolerance at the Germination Stage in Rice
Source: Front Plant Sci. 2022 Apr 8;13:887239. doi: 10.3389/fpls.2022.887239 (PMC9033254; doi:10.3389/fpls.2022.887239)
Supplement: Supplementary file 2 [file Data_Sheet_2.pdf]

Supplementary Figures

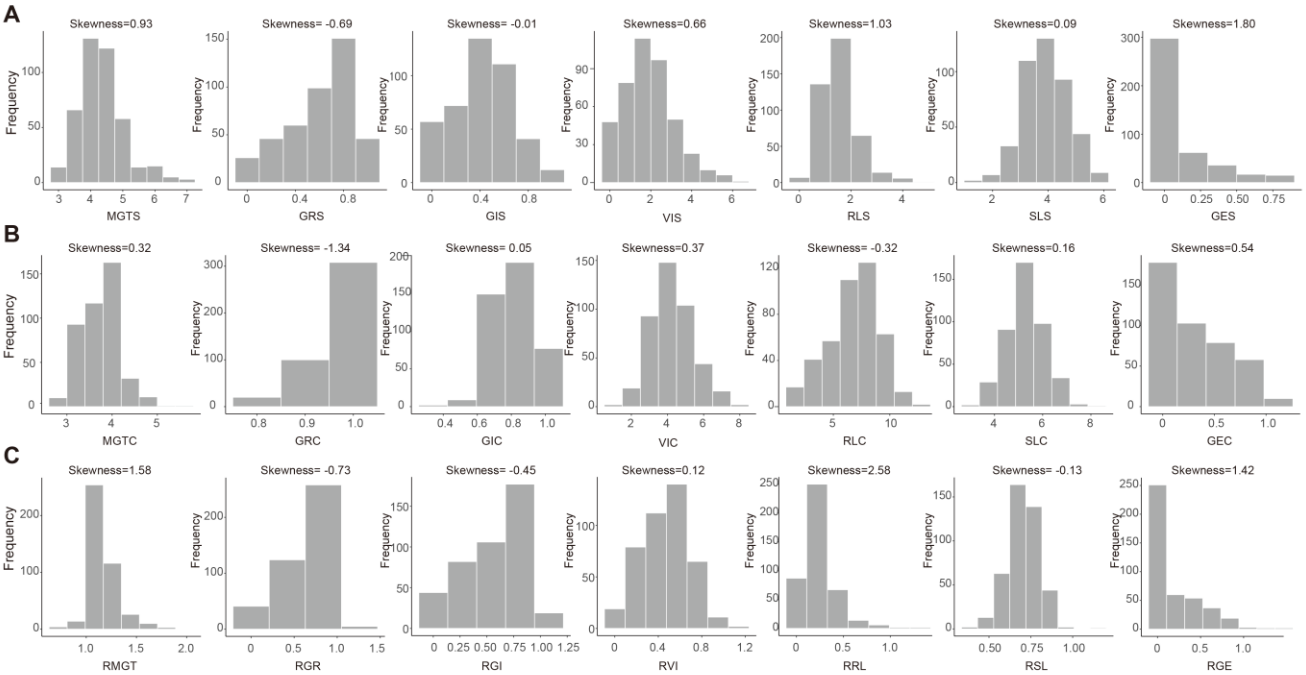

Supplementary Figure 1. Frequency distribution of the mean germination time (MGT), germination rate (GR), germination index (GI), vigor index (VI), root length (RL), shoot length (SL), germination energy (GE) for 428 rice accessions under alkali-stress and control conditions, and the relative alkali damage rate of these traits. Under alkali stress **(A)**, control **(B)**, and relative alkali damage rate **(C)**.

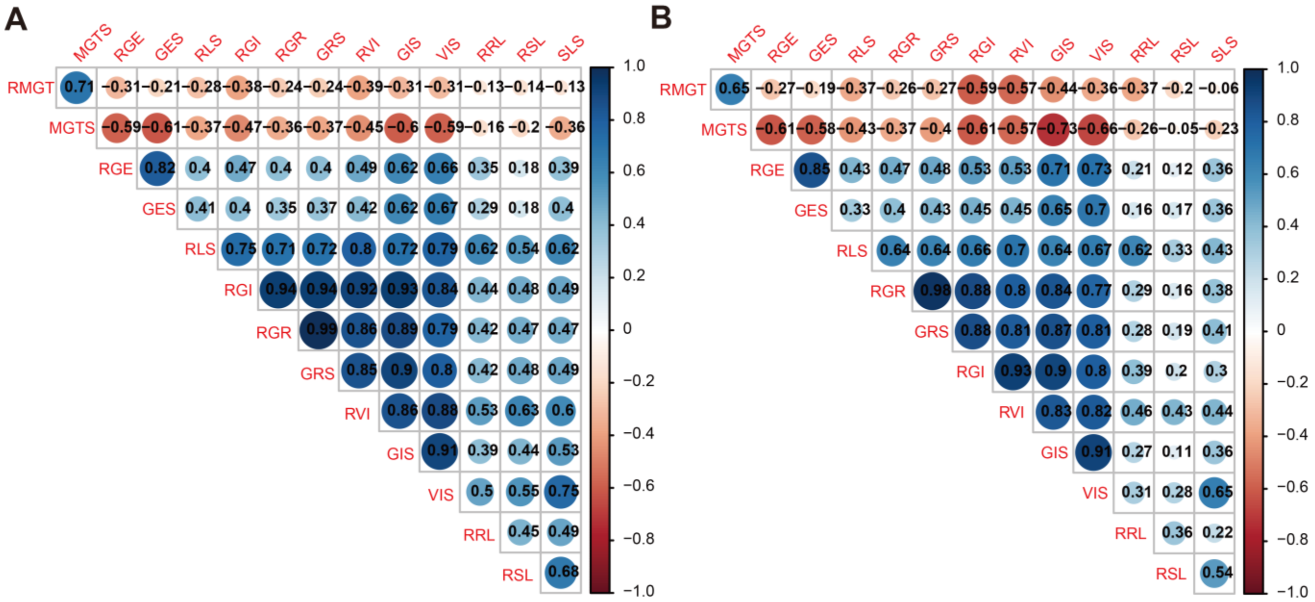

Supplementary Figure 2. Correlation coefficient of alkali-tolerance related traits. **(A)** *Xian* subpopulation. **(B)** *Geng* subpopulation.

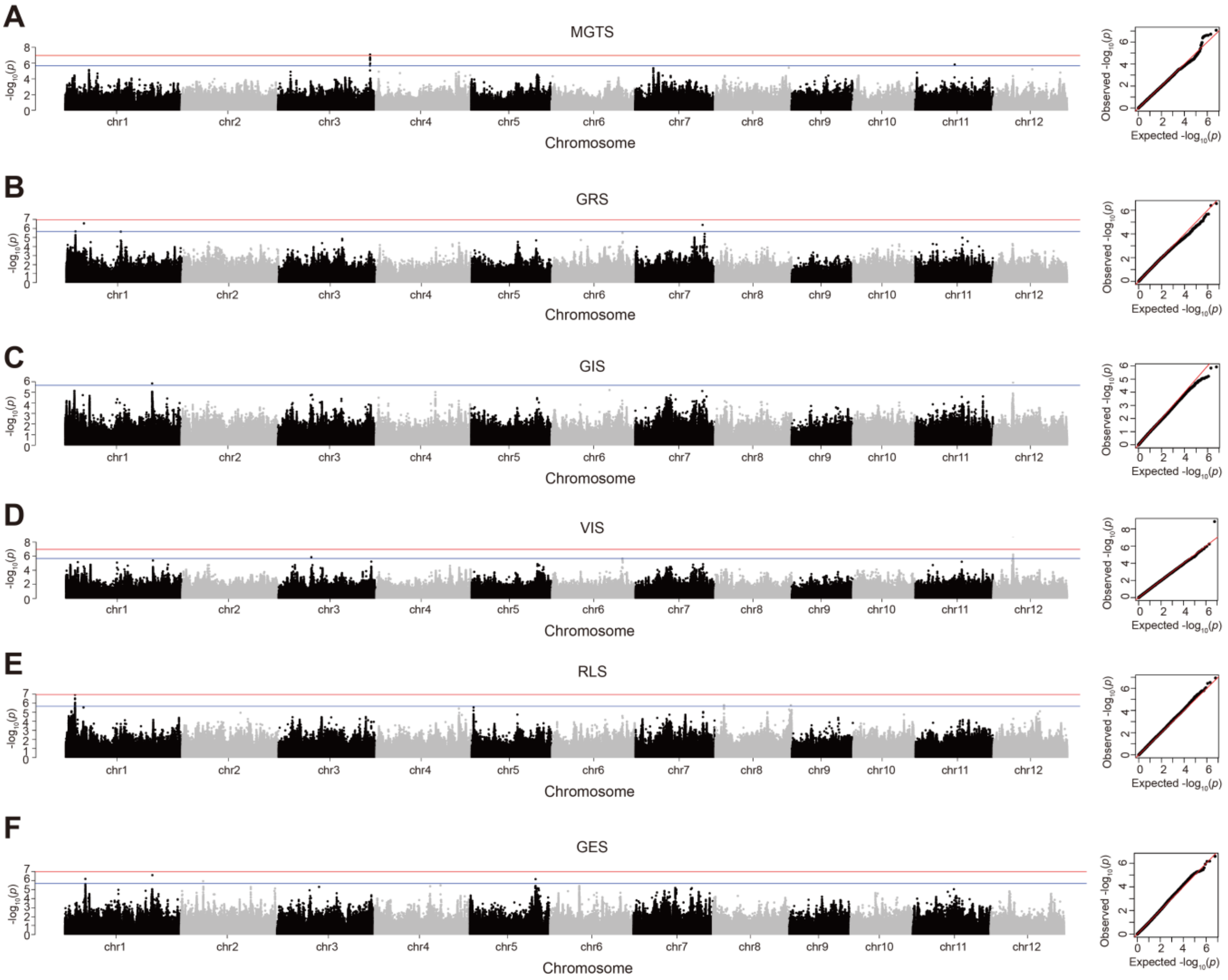

Supplementary Figure 3. Genome-wide association mapping of measured traits under alkali-stress in the whole population. Manhattan plots (left) and Q-Q plots (right) for MGTS **(A)**, GRS **(B)**, GIS **(C)**, VIS **(D)**, RLS **(E)**, and GES **(F)**. Blue and red horizontal lines indicate genome-wide suggestive and significant thresholds.

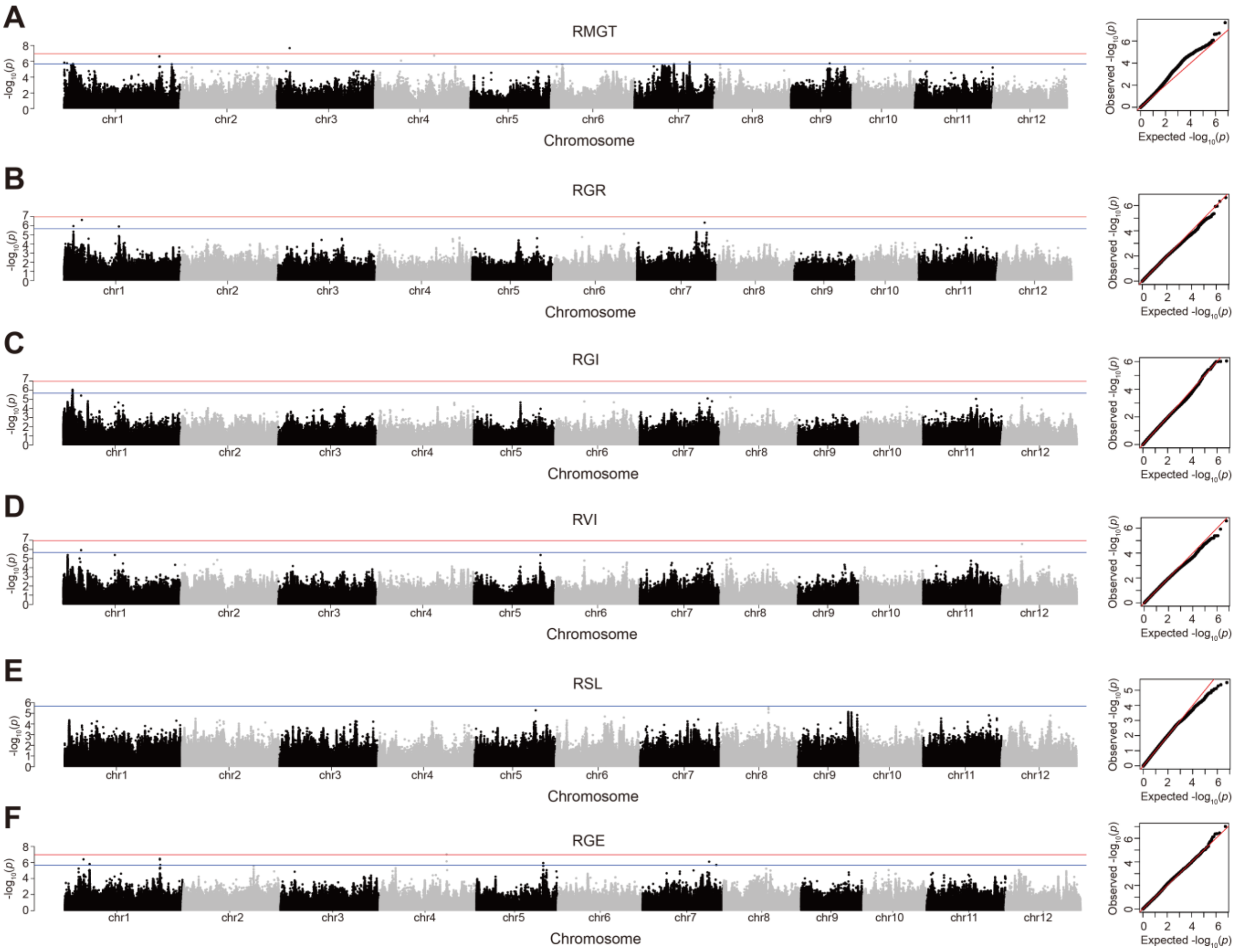

Supplementary Figure 4. Genome-wide association mapping of relative alkali damage rate of measured traits in the whole population. Manhattan plots (left) and Q-Q plots (right) for RMGT **(A)**, RGR **(B)**, RGI **(C)**, RVI **(D)**, RSL **(E)**, and RGE **(F)**. Blue and red horizontal lines indicate genome-wide suggestive and significant thresholds.

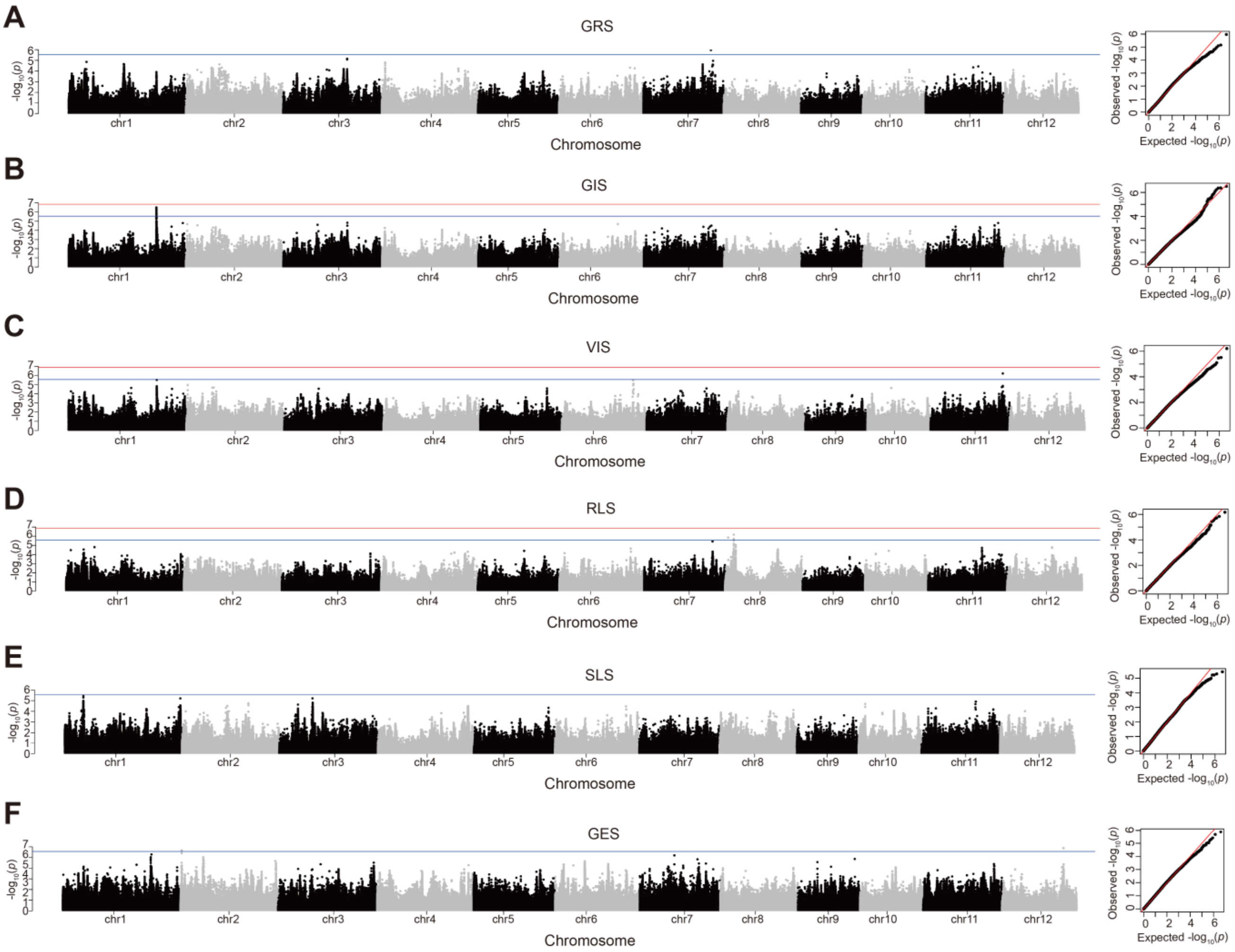

Supplementary Figure 5. Genome-wide association mapping of measured traits under alkali-stress in the *Xian* subpopulation. Manhattan plots (left) and Q-Q plots (right) for GRS **(A)**, GIS **(B)**, VIS **(C)**, RLS **(D)**, SLS **(E)**, and GES **(F)**. Blue and red horizontal lines indicate genome-wide suggestive and significant thresholds.

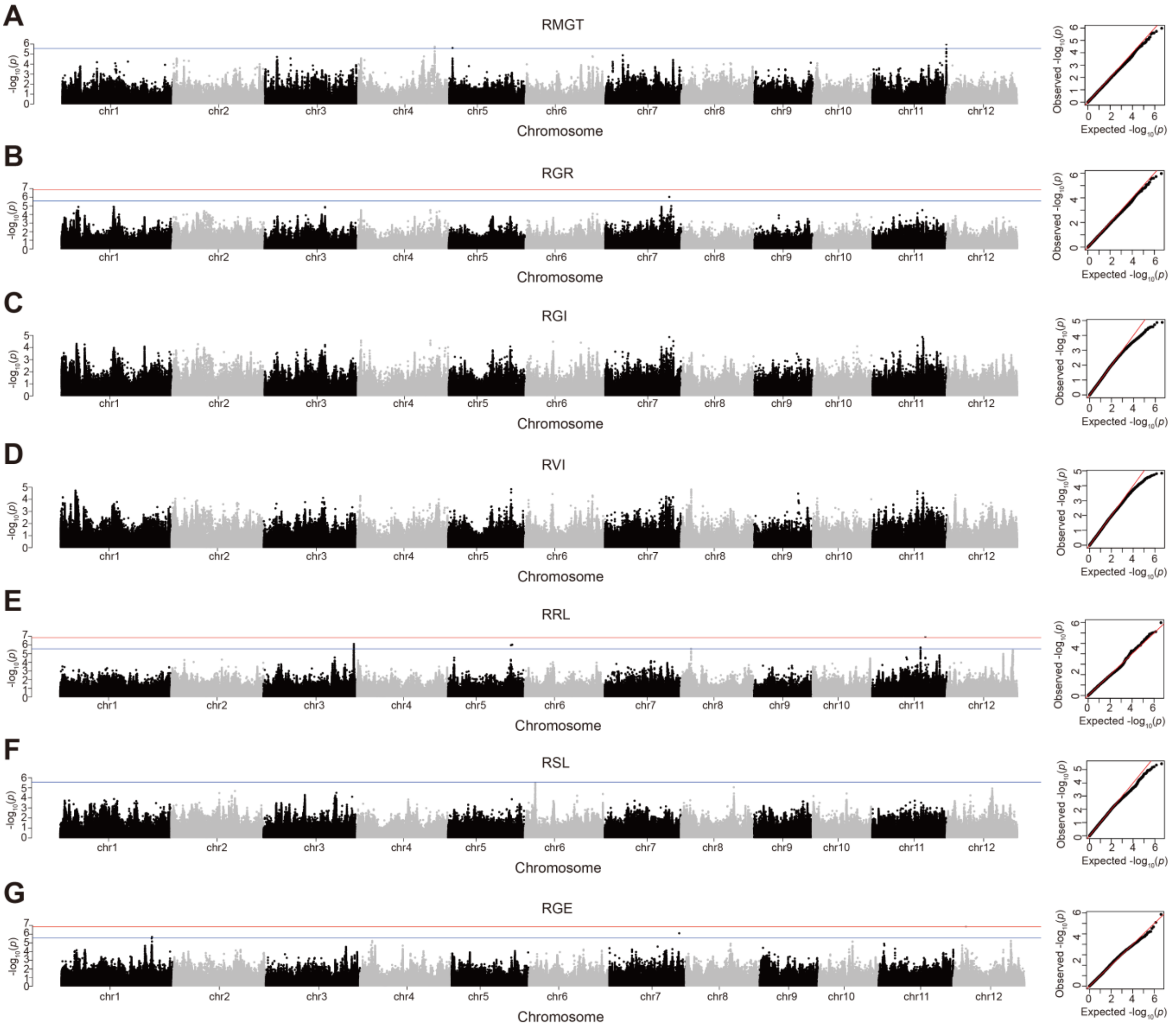

Supplementary Figure 6. Genome-wide association mapping of relative alkali damage rate of measured traits in the *Xian* subpopulation. Manhattan plots (left) and Q-Q plots (right) for RMGT **(A)**, RGR **(B)**, RGI **(C)**, RVI **(D)**, RRL **(E)**, RSL **(F)**, and RGE **(G)**. Blue and red horizontal lines indicate genome-wide suggestive and significant thresholds.

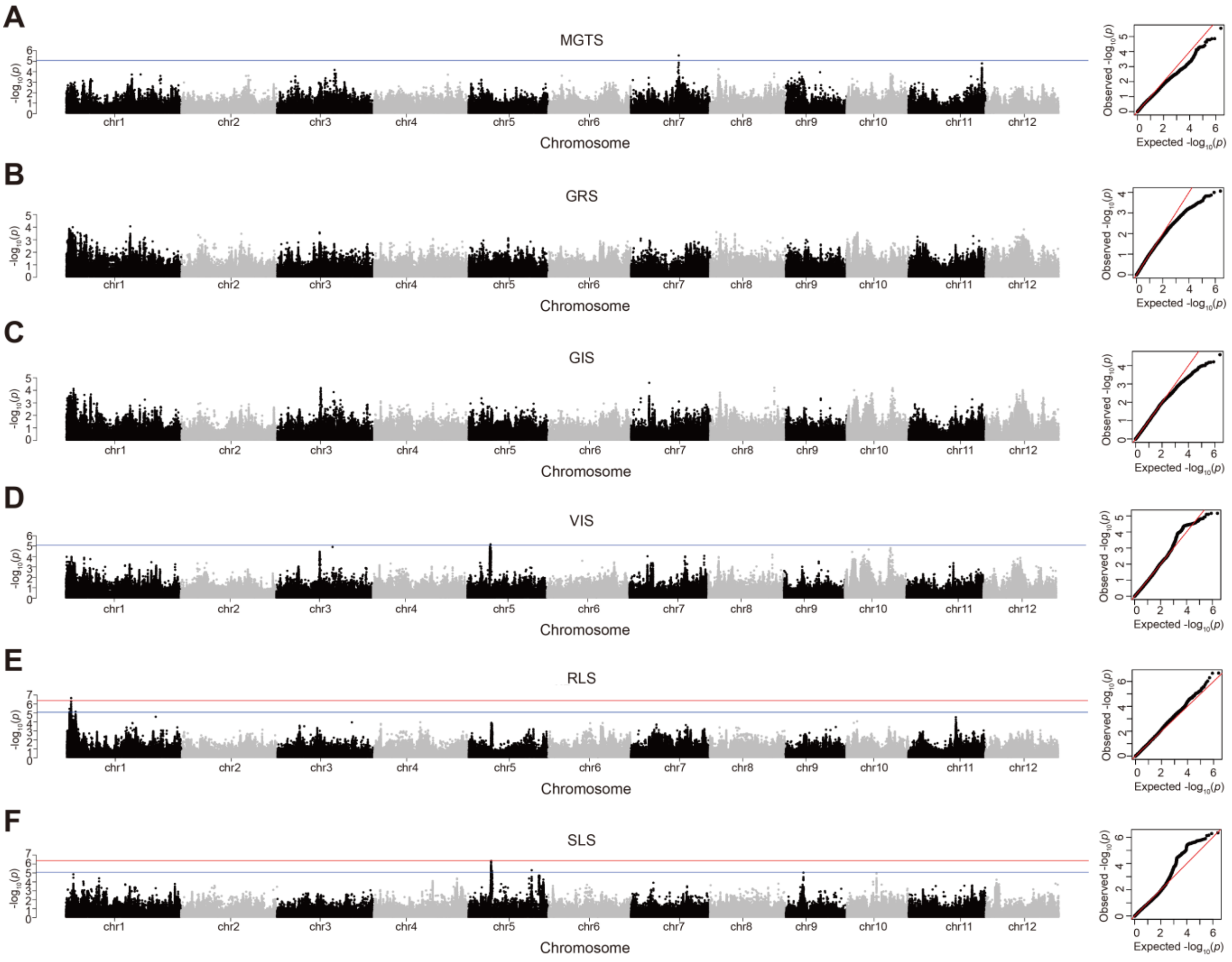

Supplementary Figure 7. Genome-wide association mapping of measured traits under alkali-stress in the *Geng* subpopulation. Manhattan plots (left) and Q-Q plots (right) for MGTS **(A)**, GRS **(B)**, GIS **(C)**, VIS **(D)**, RLS **(E)**, and SLS **(F)**. Blue and red horizontal lines indicate genome-wide suggestive and significant thresholds.

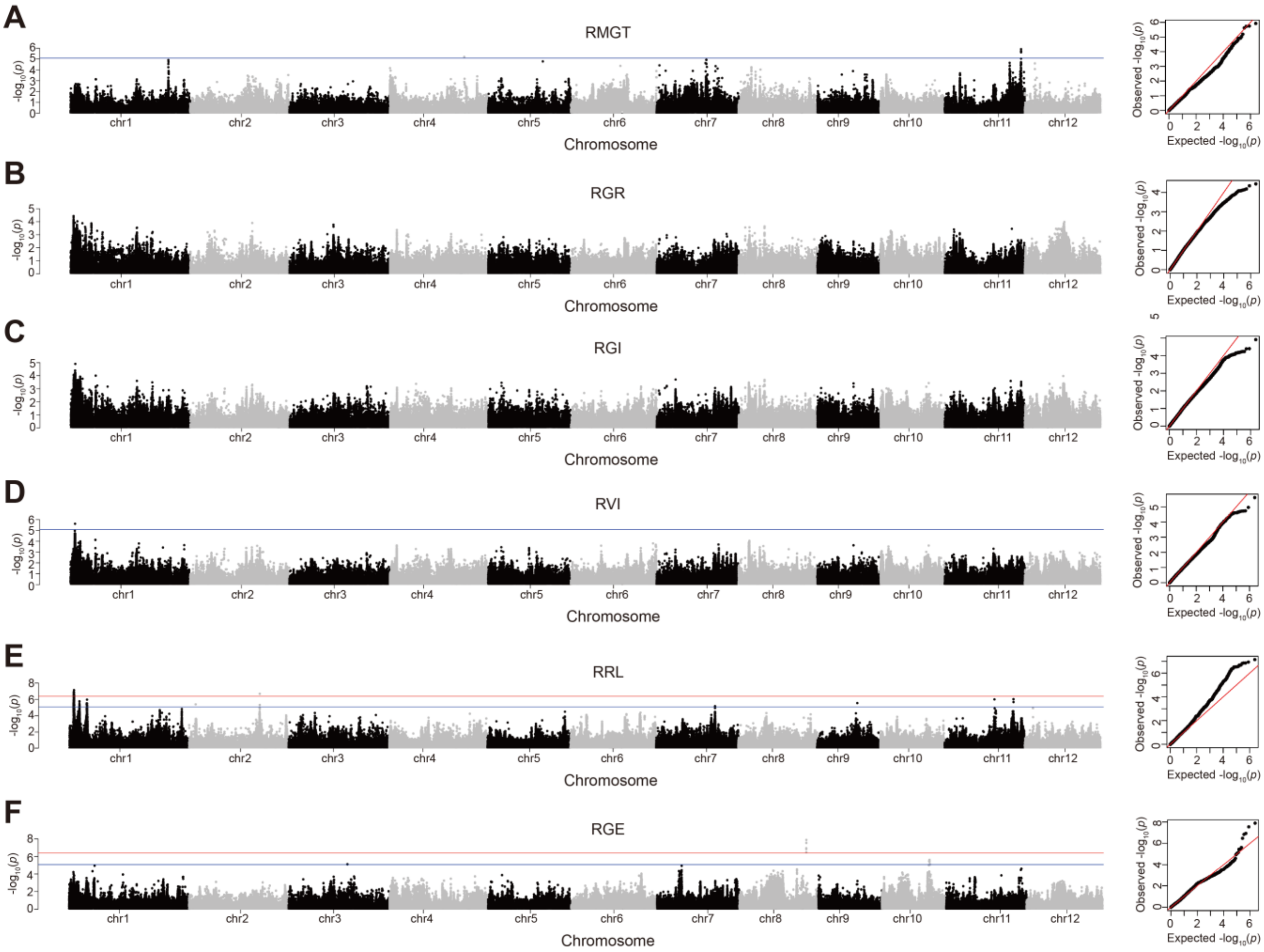

Supplementary Figure 8. Genome-wide association mapping of relative alkali damage rate of measured traits in the *Geng* subpopulation. Manhattan plots (left) and Q-Q plots (right) for RMGT **(A)**, RGR **(B)**, RGI **(C)**, RVI **(D)**, RRL **(E)**, and RGE **(F)**. Blue and red horizontal lines indicate genome-wide suggestive and significant thresholds.

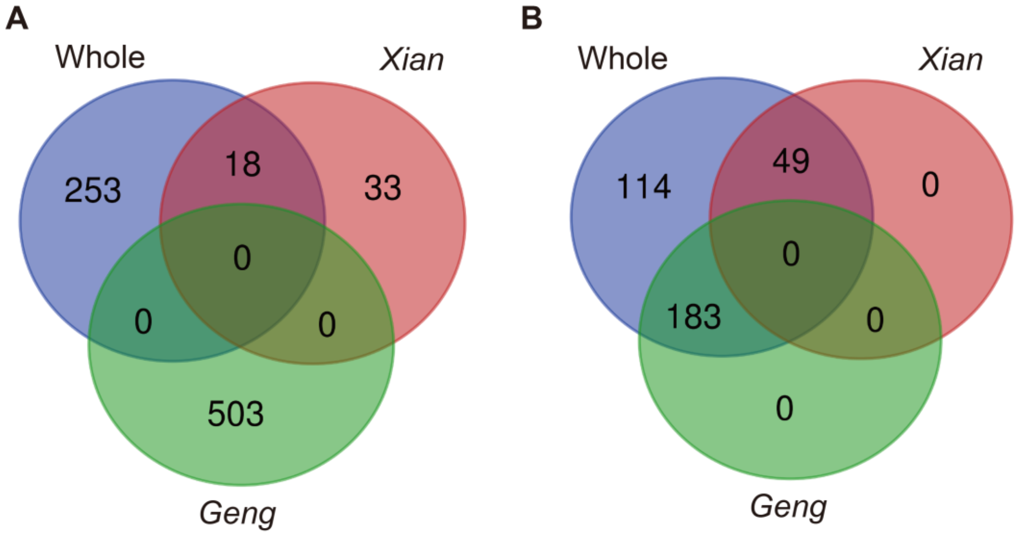

Supplementary Figure 9. Venn diagrams of association signals with alkali tolerance in the whole population, *Xian* and *Geng* subpopulations. Significantly associated SNPs **(A)** and genes **(B)**.

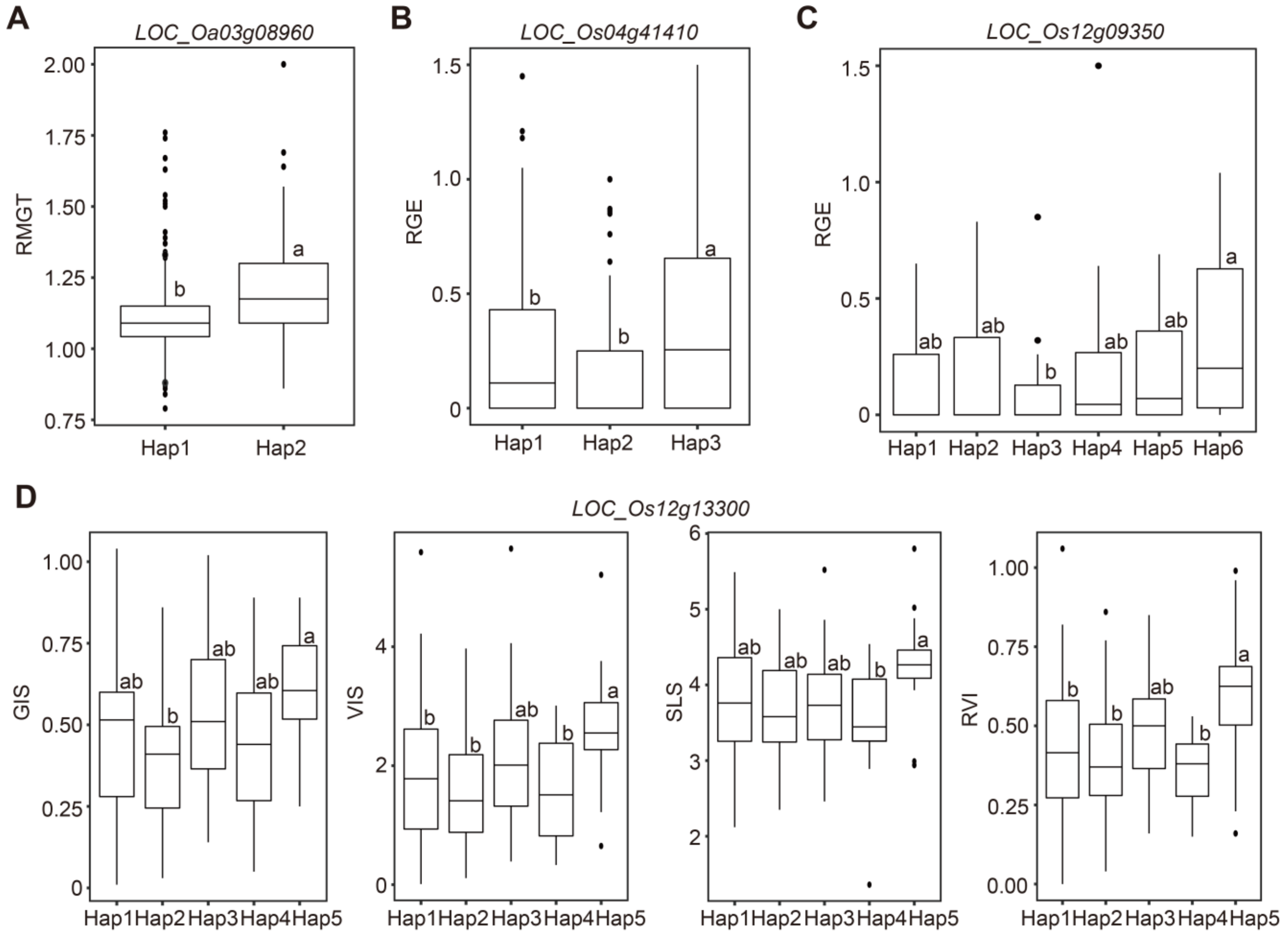

Supplementary Figure 10. Haplotype analysis of four candidate genes. **(A)** *LOC_Os03g08960*. **(B)** *LOC_Os04g41410*. **(C)** *LOC_Os12g09350*. **(D)** *LOC_Os12g13300*. Different letters above each boxplot indicate significant differences among haplotypes according to Duncan's multiple range post hoc test at $P < 0.05$.
